# Ability of extracellular volume imaging to serially measure infarct size compared to LGE within six months after acute myocardial infarction

**DOI:** 10.1186/1532-429X-17-S1-P112

**Published:** 2015-02-03

**Authors:** Martin R Sinn, Enver Tahir, Ulf K Radunski, Dennis Säring, Kai Muellerleile, Christian Stehning, Gerhard Adam, Gunnar Lund

**Affiliations:** Department of Diagnostic and Interventional Radiology, University Medical Center Hamburg-Eppendorf, Hamburg, Germany, Hamburg, Germany; Departments of Cardiology, University Hospital Eppendorf, Hamburg, Germany; Department of of Computational Neuroscience, University Medical Center Hamburg-Eppendorf, Hamburg, Germany; Philips Research Europe, Hamburg, Germany

## Background

T1 mapping and extracellular volume (ECV) imaging are promising tools to quantify increased extracellular distribution volume of contrast media after myocardial damage. We evaluated the ability of ECV imaging to measure infarct size compared to late gadolinium enhancement (LGE) after acute myocardial infarction (AMI). Additionally, the amount of cellular damage was serially measured by ECV imaging.

## Methods

CMR (1.5 Tesla Philips Achieva) was performed in 11 patients four times after reperfused AMI at baseline (BL) at 10 ±7 days after infarction and at 7.2 ±1.4 weeks (follow-up 1, FU1), 3.4 ±0.3 months (FU2) and 6.5 ±0.5 months (FU3), respectively. T1 quantification was performed before (T1pre) and 15 minutes after (T1post) administration of 0.075 mmol/kg gadolinium BOPTA on 3 short-axes for ECV calculation using the modified Look-Locker inversion-recovery (MOLLI) sequences. T1 and ECV maps were calculated with a dedicated plug-in written for the OsiriX software. Two experienced observers independently evaluated LGE-CMR as well as T1 mapping using the HeAT-Software applying a threshold method. Size of infarction and areas of prolonged post T1 or increased ECV was measured using a cutoff >2SD of remote normal myocardium.

## Results

**Figure 1:**
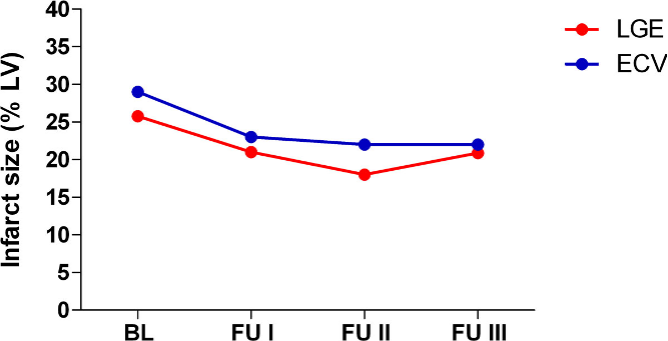
Figure 1

**Figure 2:**
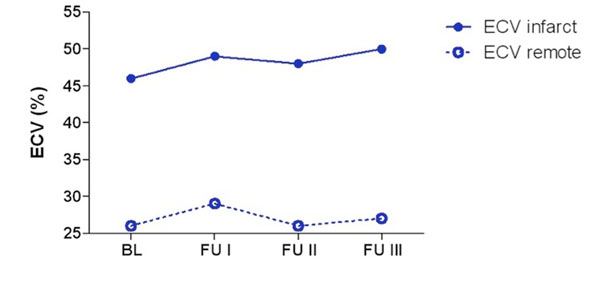
Figure 2

Infarct size on LGE images was at BL 26 ± 8%LV and decreased to 21 ±10 %LV, 18 ± 10 %LV and 21 ±9 %LV at FU1, FU2 and FU3, respectively (Figure 1). Infarct size obtained by EVC imaging was slightly, but not significantly higher with 29 ±10 %LV at BL and decreased to 23 ±8 %LV, 22 ±8 %LV and 22 ± 9%LV at FU1, FU2 and FU3, respectively (*P*=ns for all time points compared to LGE). ECV was 46±4% at BL and increased to 49±5% at FU1 (*P*<0.05) and remained constant at FU2 with 48±6% and 50±12% at FU3 (Figure 2).

## Conclusions

Similar infarct sizes were measured by LGE and ECV at four consecutive time points after AMI. ECV additionally gave information about the magnitude of cellular damage, which increased during infarct shrinkage. ECV enables to monitor infarct healing after AMI.

## Funding

This study is partially funded by the Deutsche Forschungsgemeinschaft.

